# Current Insights into the Epidemiology and Transmission Dynamics of African Swine Fever Virus and Future Control Perspectives

**DOI:** 10.3390/pathogens15060586

**Published:** 2026-05-29

**Authors:** Shanta Barua, Asep Gunawan, Autchara Kayan, Masa Tenaya, Mehmet Ulas Cinar, Made Kardena, Syeda Hasina Akter, Nurulfiza Mat Isa, Henry Annandale, Subir Sarker, David T. Williams, Sam Abraham, Jasim M. Uddin

**Affiliations:** 1 School of Veterinary Medicine, Murdoch University, Perth, WA 6150, Australia; 35606997@student.murdoch.edu.au (S.B.); hasina.akter@murdoch.edu.au (S.H.A.); henry.annandale@murdoch.edu.au (H.A.); 2 Department of Animal Production and Technology, Faculty of Animal Science, IPB University, Bogor 16680, Indonesia; agunawan@apps.ipb.ac.id; 3 Department of Animal Science, Faculty of Agriculture, Kasetsart University, Bangkok 10900, Thailand; fagrark@ku.ac.th; 4 Faculty of Veterinary Medicine, Udayana University, Bali 80234, Indonesia; masatenaya@unud.ac.id (M.T.); imadekardena@unud.ac.id (M.K.); 5 Department of Animal Science, Faculty of Agriculture, Erciyes University, 38280 Kayseri, Türkiye; mucinar@erciyes.edu.tr; 6 Department of Cell & Molecular Biology, Faculty of Biotechnology and Biomolecular Sciences, Universiti Putra Malaysia, Serdang 43400, Malaysia; nurulfiza@upm.edu.my; 7 School of Biomedical Sciences & Molecular Biology, College of Medicine and Dentistry, James Cook University, Townsville, QLD 4814, Australia; subir.sarker@jcu.edu.au; 8 Australian Institute of Tropical Health and Medicine, James Cook University, Townsville, QLD 4811, Australia; 9 CSIRO Australian Centre for Disease Preparedness (ACDP), Geelong, VIC 3220, Australia; d.williams@csiro.au; 10 School of Medical, Molecular and Forensic Sciences, Murdoch University, Perth, WA 6150, Australia; s.abraham@murdoch.edu.au; 11 Centre for Biosecurity and One Health, Harry Butler Institute, Murdoch University, Perth, WA 6150, Australia; 12 Centre for Animal Production and Health, Food Future Institute, Murdoch University, Perth, WA 6150, Australia

**Keywords:** African swine fever virus, domestic pigs, transmission, soft ticks, wild boar, biosecurity, international trade

## Abstract

African swine fever virus (ASFV) is an evolving threat to global swine health and food security, driven by its complex epidemiology, multi-host transmission cycles, and ongoing spread across countries. This review summarizes the global scenario and transmission pathways of ASFV, highlighting the outbreaks associated with evolving risk patterns to support effective disease prevention and control. ASF has been reported in pig-producing regions across Africa, Europe, and, more recently, Asia, largely driven by the spread of genotype II strains. The virus is transmitted through direct contact with infected pigs or pig-products, indirectly via contaminated materials, and through soft ticks of the genus *Ornithodoros* spp., with epidemiological patterns varying according to wildlife reservoirs and regional factors. Control measures mainly rely on early detection, movement control, strict quarantine, robust biosecurity measures, and international trade regulations. Despite significant advances, persistent challenges, including the absence of a widely available commercial vaccine, long-term stability of the virus, human activities, and inconsistencies in global response capacities, continue to hinder disease eradication efforts. This review underscores the need for transnational strategies and policies that integrate economically sustainable disease management systems and reduce the long-term impact of ASFV.

## 1. Introduction

African swine fever (ASF) is a contagious and devastating transboundary viral disease of domestic and wild pigs that impacts global pig production, international pork trade, agricultural economics, and social stability in affected regions [1,2,3]. The disease is caused by African swine fever virus (ASFV), an enveloped, double-stranded DNA virus, and is the sole member of the genus *Asfivirus* and family *Asfarviridae*, responsible for outbreaks of severe hemorrhagic disease, with mortality rates reaching up to 100% depending on the virulence of the strain [4,5]. ASFV possesses a complex multilayered virion structure and a genome of approximately 170–190 kb, encoding more than 150 viral proteins that contribute to viral replication, host interaction, and immune evasion [6,7]. Unlike many other DNA viruses, ASFV replicates primarily in the cytoplasm of infected cells, especially in monocytes and macrophages, which play a role in viral dissemination and pathogenesis [8].

Historically, the virus was first described in Kenya in the early 1920s and was largely confined to sub-Saharan Africa for several decades by maintaining a complex sylvatic transmission cycle involving wild suids such as warthogs and soft ticks of the genus *Ornithodoros* [9]. However, over the past two decades, the virus has emerged as one of the most significant transboundary diseases that threatens pig production and food security globally [10,11]. The World Organization for Animal Health (WOAH) recognizes ASF as one of the most severe transboundary animal diseases due to its rapid spread and significant threat to pig populations across international borders [12]. The introduction of genotype II into Georgia in 2007 marked the beginning of a major panzootic, which subsequently expanded across Eastern Europe, Russia, and large parts of Asia, resulting in huge economic losses in the global pork industry [11,13,14]. Since then, outbreaks of ASFV have been recorded in several countries of Europe and Asia, and more recently in Southeast Asia, the Pacific and the Caribbean, showing the ability of the virus to rapidly disseminate across continents through complex epidemiological pathways [10,15,16]. ASFV transmission occurs through multiple pathways, including direct contact between infected and susceptible pigs, indirectly through contaminated fomites and pork products, wildlife reservoirs such as wild boar, and biological vectors such as soft ticks [9,10,11,17,18].

The global spread of the virus has resulted in substantial economic losses to the swine industry, including the disruption of pork production, restrictions in international pork trade, and significant financial losses both in commercial and smallholder pig production [11]. Despite extensive research efforts, the disease continues to pose major challenges to the global pork production due to the absence of widely available safe and effective vaccines and the complex epidemiology involving domestic pigs, wildlife reservoirs, vectors, and long-time environmental persistence of the virus [11,15]. Therefore, a comprehensive understanding of the virus biology, epidemiological patterns and determinants, and transmission pathways is essential to improve and implement prevention and control strategies. This review aims to summarize the current global scenario, transmission mechanisms, and insights into the pathobiology of ASFV, highlighting the existing challenges and recent advances that may guide future research and disease control efforts.

## 2. Virus Structure, Genome and Strains

**Figure 1:**
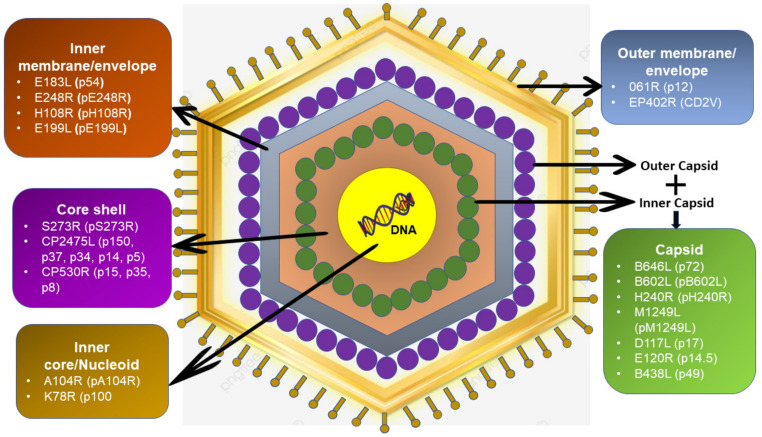
Structure of the ASF virion with different layers and their proteins.

ASFV is a large, complex DNA virus measuring approximately 200 to 300 nm in diameter [4,6,19]. The virion exhibits a highly organized, multi-layered architecture, including the outer envelope, capsid, inner envelope, core shell, and nucleoid. These layers contain approximately 151–167 open reading frames (ORFs) that encode around 150–200 viral proteins (about 68 structural proteins and more than 100 non-structural proteins) [4,19,20,21]. The complex structure of the virus leads to environmental stability and long-term persistence in the contaminated environment and animal products, which plays vital role in virus transmission and epidemiology. The structural components of the ASFV, including proteins and their functions, are summarized in Figure 1 and Table 1.

**Figure 2:**
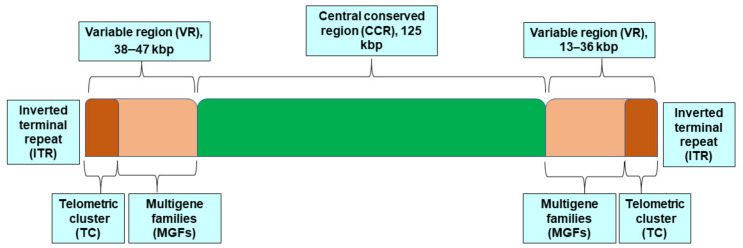
Genomic structure of ASFV.

ASFV shows significant genetic variation and evolutionary flexibility. The genome is a large linear double-stranded DNA molecule characterized by covalently closed ends that form inverted terminal repeats (ITRs) [19,20]. Many ASFV genes form multigene families (MGFs) that are arranged in tandem arrays [48,49] and play important roles in virulence, host range determination, host adaptation, and immune modulation [50,51]. Certain ASFV proteins also exhibit host-like genetic fragments; for example, the A238L protein suppresses NF-κB signaling through motifs resembling host IκB proteins, thereby contributing to virus survival and immune evasion [52,53]. The genome is organized structurally into a conserved central core region (CCR) flanked by variable regions (VR) on both terminal ends [54], where gene insertions, deletions, duplications, and mutations occur frequently [46,54,55]. These genetic changes determine the differences in virulence, transmission dynamics, and host adaptation among virus strains. The loss or acquisition of certain genomic segments and repeated sequences are responsible for strain diversity and viral evolution over time [55]. In contrast, the CCR remains conserved and is often used as an epidemiological marker for genotype differentiation of ASFV isolates [56,57]. Several genome sequence analyses have identified single-nucleotide polymorphisms (SNPs) and gene rearrangements that are useful for tracing the origin and evolution of specific outbreaks [57,58]. Arrangement of the genomic structure of ASFV is shown in Figure 2.

ASFV isolates have traditionally been classified into 24–25 genotypes based on sequence variation in the C-terminal region of the B646L gene encoding the p72 capsid protein [59,60]. However, recent genomic re-evaluation has suggested that these historical genotypes can be consolidated into six major p72 groups, emphasizing the need for more robust whole-genome-based classification approaches [60]. Among these, genotype II is considered the most significant as it is responsible for the major global outbreaks that started in Georgia in 2007 and subsequently spread across Europe and Asia [61]. In addition, molecular markers such as the O174L gene have been used to distinguish closely related strains and to track the origin of outbreaks, such as those reported in Poland [62]. Recent genomic research continues to reveal emerging ASFV variants and regional lineages. For instance, genome sequencing of ASFV outbreaks in West Africa revealed two main viral lineages circulating in Nigeria, indicating that the virus is still evolving and diversifying in different regions [58]. In Europe, a multigene typing approach has identified at least 26 distinct variants of genotype II ASFV [63,64]. In addition, several countries have reported variants with genetic deletions or reduced virulence, proving that ASFV continues to adapt to changes in the interfaces between its environment and host populations [65,66]. This high level of genetic variation creates challenges for vaccine development, accurate diagnosis, and effective disease control [67].

## 3. Pathobiology, Transmission, and the Global Situation

### 3.1. Pathobiology of ASFV

Primary replication of the virus starts in the macrophages of the oropharyngeal region, including tonsils and regional lymph nodes [67,68]. The virus subsequently spreads to key organs, including the spleen, bone marrow, liver, lungs, and kidneys through the bloodstream, causing viremia [69,70]. Despite primary viral replication in the macrophages, infection of endothelial cells and other cell types has also been observed [68,70]. Although the precise host receptors remain incompletely defined, the virus enters host cells through different pathways, including micropinocytosis, clathrin-mediated endocytosis (CME), phagocytosis, involvement of Fc-receptor, apoptotic mimicry, and utilization of apoptotic bodies [71]. A distinctive feature of the virus is that it replicates within specialized ‘viral factories’ in the cytoplasm of the infected cells [50,68]. Viral structural proteins contribute to attachment to the host cell surface and promote uptake into the target cell, whereas viral genomic nucleic acid can alter macrophage phagocytic activities, further influencing the host defense mechanism for successful replication [67]. Many proteins of the virus manipulate host cellular pathways to facilitate viral replication and spread by suppressing antiviral interferon responses [72,73].

ASFV evades the host defense mechanisms in different ways. It applies immunomodulatory strategies to evade early host defenses to establish infection and promote disease [68]. These mechanisms include suppressing the signaling of type I interferon (IFN), modulating antigen presentation, and interfering with cytokine networks. Recent studies indicate that infection with ASFV activates cytoplasmic DNA-sensing mechanisms, including the cGAS-STING pathway, leading to downstream signaling that may simultaneously amplify proinflammatory responses while limiting effective antiviral immunity [40,74]. ASFV also interferes with how infected macrophages process and present antigens, which makes it harder for the adaptive immune system to become fully activated. Additionally, the virus leads to the reduced production of cytokines by altering the NF-κB and JAK-STAT signaling pathways [75,76]. This weakens the host antiviral defenses and contributes to immune dysregulation and facilitates systemic disease progression.

Infected macrophages produce high levels of proinflammatory cytokines, including IL-1, IL-6, TNF-α, driving severe systematic inflammation and tissue injury through contributing to a cytokine storm [50,77]. Inflammatory mediators such as TNF-α and IL-1β exaggerate acute ASF disease by causing vascular injury, tissue edema, and organ dysfunction [69]. The virus initially inhibits apoptosis in infected cells to allow viral replication; however, later, extensive apoptosis of uninfected lymphocytes through immune signaling results in lymphoid depletion and immune collapse [41,70]. Moreover, the virus causes pathological changes in the vascular endothelium. Endothelial dysfunction, platelet abnormalities, and coagulation cascade disturbances lead to disseminated intravascular coagulation (DIC), as well as widespread hemorrhage observed in acute infection [68,69]. Since ASFV affects the key innate immune cells in pigs, it causes significant changes in the host immune response and pathobiological changes, leading to severe disease outcomes and high mortality.

### 3.2. Transmission Mechanisms

ASFV transmission is multifaceted, including direct and indirect transmission across farm environments, between domestic and wild pig populations, and via arthropod vectors. The primary route of transmission is direct contact between infected and susceptible pigs through oro-nasal contact or contaminated bodily fluids, respiratory secretions, and excreta containing high viral loads [78,79]. In addition, field investigation suggested that short distance airborne spread through droplets or contaminated dust may infect pigs in adjacent piggeries even without direct contact [12]. These observations show the importance of environmental factors and housing conditions in the spread of the virus.

Indirect transmission plays a major role in the epidemiology of ASF. Contaminated fomites such as farm equipment, vehicles, clothing, footwear, and veterinary instruments serve as mechanical carriers, facilitating virus transmission between pens and farms [12]. Unrestricted personnel movement between premises without adequate disinfection and biosecurity measures exacerbate virus dispersal [80]. Moreover, the virus remains infective for prolonged periods in raw or uncooked pork products, enabling infection of naïve herds through contaminated swill feed or water [81,82].

The disease dynamics could be further complicated by the involvement of wild suids in the epidemiological landscape of ASF. In many parts of Europe and Asia, wild boar populations act as significant reservoirs, maintaining ongoing viral circulation in the environment and serving as infection sources for neighboring domestic pig herds through overlapping habitats, shared water bodies or feed sources, especially in backyard or outdoor production systems or low-biosecurity settings [83,84,85]. In addition, carcasses of infected wild boar contribute to local transmission cycles via environmental contamination and scavenging by other wild boar or interaction with virus-contaminated soil and vegetation [86].

In endemic regions of sub-Saharan Africa, vector-mediated transmission has been well documented. The sylvatic transmission cycle is maintained, where soft-ticks (genus: *Ornithodoros*) interact with wildlife reservoir hosts, such as warthogs (*Phacochoerus africanus*), bushpigs (*Potamochoerus larvatus*), red river hogs (*Potamochoerus porcus*), and giant forest hogs (*Hylochoerus meinertzhangeni*), and facilitate virus circulation between wild suids and domestic pigs [87,88]. Soft ticks such as *O. moubata complex* in Africa and *O. erraticus* in Europe acquire the virus during blood feeding and subsequently transmit it through bites, maintaining long-term tick–pig transmission cycles through transstadial and transovarial transmission [5]. The virus replicates in various organs and body parts of the ticks and persists in a viable state for extended periods, ranging from 23 to 239 days, depending on the species. Even longer survival times have been documented, reaching up to 3 years in *O. moubata*, 5 years in *O. erraticus*, and over 500 days in *O. coriaceus* [79,89,90]. The first investigation of localization of ASFV in *O. porcinus* was conducted by Rock [89], showing that the virus multiplies primarily in hemocytes (types I and II), followed by secondary replication sites such as tissues in the midgut epithelium, phagocytic cells, connective tissues, salivary glands, coxal glands, and reproductive organs. The peak viral titers were recorded after 91 days of infection in the salivary glands and reproductive tissues [90]. Consequently, infected ticks can potentially act as long-term reservoirs by protecting the virus from environmental stress and sources of reinfection, whereas long-distance transmission is mediated indirectly through the movement of hosts carrying infected ticks and contaminated materials [79]. In addition, virus survival in the ticks, even in the absence of hosts, complicates the eradication process. These features make ASFV a unique DNA arbovirus transmitted by arthropod vectors [91]. Moreover, mechanical transmission by certain hematophagous insects, such as the stable flies (*Stomoxys calcitrans*), has been demonstrated experimentally, although their epidemiological significance in natural outbreaks is less studied [92].

**Figure 3:**
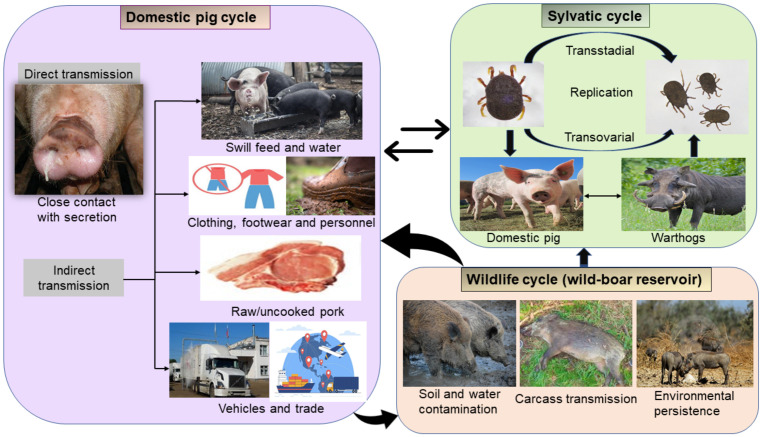
Transmission dynamics of ASFV.

The complex transmission dynamics of ASFV highlight the importance of adopting integrated perspectives targeting direct contact, environmental stability, and indirect spread via vectors, fomites, and wildlife-livestock interfaces. Effective control strategies therefore require coordinated biosecurity, surveillance, monitoring, and management practices to mitigate virus spread within and between pig populations. The different transmission dynamics of ASFV are illustrated in Figure 3.

### 3.3. History and Global Distribution

Although ASF outbreaks have been occurring since 1909, the virus was first formally documented in 1921 as a severe, highly fatal hemorrhagic disease affecting pigs owned by European settlers in Kenya, East Africa [68,93,94,95]. Comparable cases have also been observed earlier in Zambia’s Eastern Province since 1912 [96]. During the late 1920s and early 1930s, reports of ‘East African swine fever’ were noted in the north-eastern regions of South Africa, and subsequently confirmed the disease in Angola and Malawi [97,98]. By the mid-1990s, the disease persisted as a wildlife-associated (sylvatic) infection across much of eastern and southern Africa, extending into the southern Central Africa [99]. Its maintenance in these regions was linked to a natural transmission cycle involving warthogs and *Ornithodoros* ticks [9,17].

From 1930 to 1950, the virus persisted endemically in sub-Saharan Africa, where multiple genotypes evolved and circulated between domestic pigs and wildlife, periodically causing severe outbreaks in smallholder systems [98,100]. In the late 1950s, the virus spread into Europe and West Africa, marking its first transcontinental incursions [10]. ASFV persistence in Africa is primarily driven by wildlife–domestic interfaces rather than trade networks. In the future, risk mitigation efforts in Africa should therefore focus on controlling wildlife–livestock interfaces, strengthening smallholder biosecurity, and enabling early detection in high-risk ecological zones.

In Europe, ASFV was first detected in Portugal in 1957, likely introduced through contaminated pork products [10,101]. Although the initial outbreak was rapidly controlled, a reintroduction in 1960 led to sustained transmission across the Iberian Peninsula, particularly in Portugal and Spain, where the disease persisted for more than three decades and demonstrated significant transboundary and long-distance dissemination [102,103]. Outbreaks were also reported in several western [10,17,101,103] and eastern European countries [104] (Table S2), but eradication programs successfully eliminated the virus by the mid-1990s. However, ASF remained endemic on the island of Sardinia, following its introduction in 1978, for more than four decades, until intensive eradication and biosecurity programs led to official declaration of being ASF free in 2024 [105,106,107]. Thus, situations in Europe indicate remarkable epidemiological adaptability and transboundary transmission potential of the virus, emphasizing the importance of biosecurity and eradication programs to mitigate the risk of future outbreaks and endemic persistence.

In 2007, genotype II of ASFV was introduced into Georgia through contaminated pork products [4,13]. This pivotal epidemiological shift marked the beginning of the contemporary Eurasian panzootic. The virus established an endemic cycle in wild boar and domestic pigs and spread across the Russian Federation and Eastern Europe from the Caucasus [108]. It subsequently reached the Baltic States, Poland, Germany, and other European Union countries, where factors such as wild boar density, carcass persistence, and human-mediated movements contributed significantly in viral spread [109,110]. However, the dominant transmission pathways varied regionally; wildlife-driven transmission and environmental persistence predominated in eastern Europe, whereas human-mediated activities such as illegal pork trade, contaminated vehicles, and fomites were considered to initiate new outbreaks in western Europe [111]. Phylogenetic analysis confirms that most recent European outbreaks are linked to genotype II lineages derived from the 2007 introduction, and these strains have gradually diversified due to repeated transboundary movement [112]. Overall, ASFV persistence and expansion in Europe reflects a complex interplay between wildlife ecology and anthropogenic factors.

In 2018, the epidemiological landscape changed dramatically with the confirmation of ASFV in the world’s largest pig population, China [113,114]. As a result of the long-distance transport of pigs and pork products, weak biosecurity, and the complex production network, the virus spread extensively across Chinese provinces within months [115]. The virus subsequently expanded across east and southeast Asia, including Vietnam, where widespread outbreaks from 2019 to 2024 resulted in substantial herd losses and restructuring of the pig farming sector [116]. Similar genotype II outbreaks were reported in the Philippines, Indonesia, Timor-Leste and other parts of the region [70,117]. Whole-genome sequencing of the virus in Indonesia revealed close relatedness to other Asian genotype II strains, indicating regional connectivity [16]. South Asia experienced its first confirmed outbreak in India in 2020, which further expanded southward contributing to the Asian epidemic spread including Nepal and Sri Lanka [113,118,119]. Therefore, ASFV control in Asia cannot rely solely on biosecurity; instead, it requires strengthened regional trade surveillance, border security enforcement, and coordinated transboundary monitoring systems to establish effective regional containment.

Since its first description, ASF has persisted in Africa for more than a century through the complex interface of wildlife reservoirs, tick vectors, and low-biosecurity pig production systems [9,58,120]. In contrast, the Americas remained largely ASF-free after extensive eradication efforts in the 1980s. However, re-detection in the Dominican Republic and Haiti in 2021 highlighted the propensity for ASFV to re-emerge and emphasized the importance of robust surveillance systems and effective control measures [121]. Although the United States is still ASF-free, modelling studies suggest that an outbreak could cause major economic losses due to the large pig industry and export market of the country [122]. Therefore, strengthening cross-border surveillance, improving biosecurity in smallholder systems, and enhancing regional collaboration remain critical for preventing further spread of ASFV.

To date, Australia remains free from ASFV; however, its proximity to Southeast Asia, strong international trade connections, and presence of the virus in northern neighboring countries, including Timor-Leste, western Indonesia and Papua New Guinea, create a persistent incursion risk [16,123,124]. Economic assessments estimate that a small-scale outbreak of ASFV in Australia could result in losses of $117–263 million in domestic pigs, with eradication in feral pigs costing approximately $101–127 million, whereas endemic establishment could incur around $0.4–2.5 billion [125]. Risk assessments and fuzzy modelling approaches identified possible pathways, including illegal pork import, swill feeding, contaminated fomites, and international passenger movement [126]. Within Australia, higher outbreak risk is predicted in regions with high-pig density and a large overlapping population of feral pigs [127,128]. In addition, temperature and rainfall patterns could influence the virus survivability and spatial spread [129]. Although direct Australian evidence remains limited, international studies suggest that vehicles used in livestock transport, shared equipment, and long-distance pig movements could facilitate farm to farm transmission [130]. This emphasizes the importance of strict cleaning and disinfection protocols and effective traceability systems to reduce transmission risk [131].

**Figure 4:**
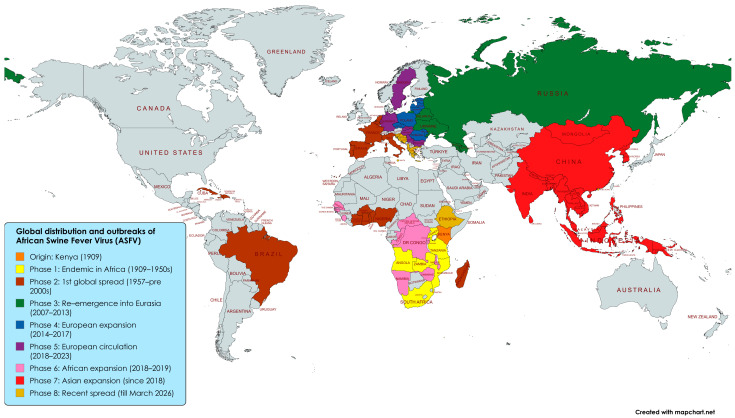
Global distribution and outbreaks of ASFV from 1909 to 2026.

As of 2025–2026, continuous circulation and sporadic new outbreaks have been reported in domestic pigs and wild boar across parts of eastern Europe, southeast Asia, and sub-Saharan Africa [95,132]. Nearly 2000 cases were detected both in domestic pigs and wild boar over 16 European between January and February 2026 [133]. The UK has also been identified as high-risk due to human-mediated pathways, wild boar movements, and non-commercial pork imports [133]. Genotype II remains the dominant ASFV strain circulating across Eurasia, although whole-genome and multi-gene sequencing studies are now showing increasing genetic variation [63,64,112]. Despite advances in genomic epidemiology and outbreak modelling, the disease remains difficult to eradicate once established in wild pig populations, especially in areas with inconsistent biosecurity and limited cross-border coordination [68]. Global distribution and sequential outbreaks of ASFV are shown in Figure 4 and Supplementary Table S1.

ASFV infection in pigs can occur simultaneously with other viral infections, especially where multiple swine diseases circulate. For instance, one of the earliest cases of co-infection of ASFV with porcine circovirus type-2 (PCV-2) was documented in pigs from Indonesia and Mongolia [134]. As PCV-2 infection can induce immunosuppression, such co-infections may elevate disease severity through increasing susceptibility to secondary pathogens during ASF outbreaks. In West Africa, genomic screening of pigs in Nigeria revealed that ASFV-infected pigs frequently harbored multiple viral agents simultaneously, including PCV-2, PCV-3, and porcine parvovirus-1 (PPV-1), with nearly half of the sampled pigs testing positive for two or more pathogens [135]. Additionally, diagnostic studies have shown the possibility of concurrent infection of ASFV with classical swine fever virus (CSFV) in endemic regions, emphasizing the importance of multiplex molecular assays to differentiate these clinically similar infections [136]. These findings suggest that ASF outbreaks often occur in complex pathogen environments, complicating clinical diagnosis, surveillance, and control strategies in affected pig populations.

## 4. Key Determinants of ASFV Epidemiology

A key determinant in ASF epidemiology is the remarkable environmental stability of ASFV, which enables prolonged survival in pork products, carcasses, and contaminated fomites [8,105]. Persistence of the virus in organic materials, including blood and tissues, enhances exposure through contaminated equipment, clothing, vehicles, feed, and water, contributing significantly to farm-to-farm spread [1].

Lack of biosecurity and management practices are major risk factors for ASF outbreaks in both commercial and backyard production systems. Epidemiological investigation in the Philippines demonstrated that the absence of perimeter fencing, poor visitor control measures, insufficient vehicle disinfection, and limited quarantine protocols significantly increased the risk of virus introduction into domestic pig farms [137]. Swill feeding, particularly the use of improperly treated food waste containing infectious pork products, remains a historically documented and epidemiologically significant risk factor, especially in smallholder or backyard systems [138]. Furthermore, a lack of biosecure feed sourcing, inadequate carcass disposal, deficiencies in cleaning and disinfection, improper manure and effluent management, and environmental contamination of water sources are linked to higher disease outbreaks [1,138,139,140].

Host population and husbandry practices also increase disease transmission risk [141]. High pig density accelerates virus spread once introduced within the herd. In smallholder or backyard farming systems, pigs are often reared under low input conditions with limited confinement and biosecurity, particularly in free-roaming pig systems, which increases the probability of contact with neighboring herds or wildlife [140,141]. Virus amplification and transmission may also be facilitated by mixed production systems where animals of different ages and health status are reared without strict segregation rules or practices [142].

Governance factors and socio-economic conditions also contribute substantially to the risk of ASF. Prolonged outbreak duration and wider dissemination of the virus have been noted in regions with limited veterinary infrastructure, delayed reporting systems, and insufficient compensation schemes. The transmission pathways are also influenced by informal trade networks and pig slaughter and pork distribution practices [80,143]. Global ASF studies further emphasize that farmer awareness, monitoring systems, and compliance with control measures strongly influence epidemic outcomes [1].

**Figure 5:**
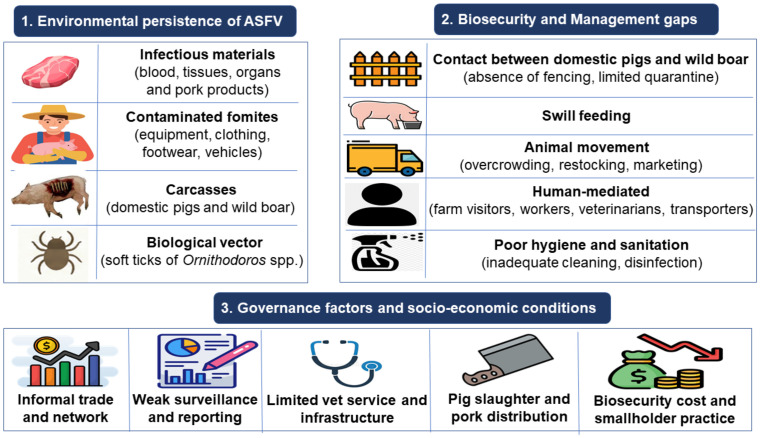
Key determinants influencing the spread of ASFV in domestic pig herds.

The risk profile of ASF reflects the complex interactions of several factors, including viral persistence, host density, wildlife ecology, farm biosecurity, socio-economic conditions, and trade networks. For designing evidence-based control programs and further preventing geographical expansion of the disease, a comprehensive understanding of the interconnection of these risk factors is essential. Therefore, an integrated approach combining strengthened biosecurity, controlled animal movement, wildlife management, improved surveillance systems, and community engagement are required for effective mitigation strategies of the disease. The key determinants that influence ASFV dissemination are demonstrated in Figure 5.

## 5. ASFV Control and Management Strategies

The dissemination of ASF can be controlled only through the prompt detection and implementation of standard disease management practices, such as ongoing surveillance, epidemiological assessments, tracing of pig movements, stamping out of infected populations, enforcing biosecurity protocols, applying quarantine measures, and the regulation of animal movement [144].

### 5.1. Infection Management and Containment

Once an outbreak of ASF occurs, infection management becomes challenging. In an ideal scenario, outbreak containment relies on rapid diagnosis and immediate notification to the veterinary authorities to enable emergency response activities [8,145]. Following confirmation, movement restrictions on pigs are enforced, and zoning systems comprising infected, surveillance, and protection zones are established to prevent further disease spread [145]. Furthermore, safe carcass disposal, the destruction of contaminated animal products, thorough cleaning and disinfection of infected premises, restriction of the movement of farm personnel and contaminated equipment, and epidemiological tracing systems should be implemented to identify infection sources and guide targeted interventions [145]. Since commercially available vaccines are limited, a combination of these measures is essential for rapid containment to prevent regional spread [8]. However, due to the high pathogenic nature of the virus and its severe economic impact, stamping out at the herd level remains the preferred strategy in ASF-free countries.

### 5.2. Culling and Depopulation

Culling and depopulation are widely used control measures to limit the transmission of ASFV once detected in domestic pig populations. The rapid removal of infected animals and separating exposed pigs can help halt within-and between-farm spread [2], whereas whole-herd depopulation has traditionally been applied to quickly eliminate infection sources and reduce further dissemination risk [146]. However, this approach may lead to major economic losses, particularly in large commercial pig industries and in countries with limited or no compensation.

Socio-economic conditions and farming practices are key considerations in the implementation of culling and depopulation. Recent studies emphasized alternative approaches such as selective or partial culling [147,148,149]. Studies conducted in Vietnam showed that partial culling involving removal of only infected and high-risk contact animals and selective culling in combination with strict monitoring, quarantine of affected pens, and improved hygiene measures act as potential effective approaches for managing ASF outbreaks [116,147,148]. Similar studies in China reported that precise culling, along with regular testing and strict movement control, helped to eliminate the virus while protecting unaffected pigs [149]. These findings suggest that targeted depopulation strategies may be effective when early detection systems and strong biosecurity are applied.

In wildlife populations, culling has also been used to reduce disease reservoirs. For example, in Europe, intensive carcass removal combined with hunting has been suggested as an effective strategy to reduce virus persistence in wild boar populations [150]. Furthermore, targeted culling of infected wildlife population can slow down the spread of the disease, although the success rate depends on population density, surveillance capacity, and landscape factors [151]. Overall, culling and depopulation remain essential components of ASF outbreak control; however, this should be applied very carefully and together with other control strategies.

Cost sharing is critical for effective culling or depopulation during ASF outbreaks as it distributes the financial burden between government and industry, enabling rapid response and compliance. For instance, in Animal Health Australia’s Emergency Animal Disease Response Agreement (EADRA), the costs of the outbreak response, including culling and compensation, are jointly funded with the aim of improving early reporting and timely eradication [152,153]. Although private livestock insurance exists, government compensation schemes are the primary mechanisms to support depopulation and helps farmers restock rather than rely solely on insurance [152]. For example, Australia provides compensation (market-value payment) for animals destroyed under official disease control and cost is shared under the EADRA framework. In contrast, in endemic regions of Africa and Asia, the lack of compensation has led to emergency sales and the informal slaughtering of infected pigs locally by small holders or the improper disposal of infected carcasses, leading to under-reporting and ‘silent spread’ of the virus into the market value chain [154,155,156]. Nevertheless, prevention is better as depopulation is impossible for many countries considering the economic condition of the farmer and the lack of financial support or insurance.

### 5.3. Vaccination

ASF vaccine development has been a high priority for decades but has gained further impetus in the last 20 years with the emergence of ASFV in Europe and Asia. Although first generation vaccines have recently been commercialized, their approval and use are limited to certain countries. Therefore, disease control still largely relies on strict biosecurity, surveillance, and the culling of infected animals. However, the rapid global spread of the disease since the late 2000s has increased the importance of developing effective vaccines capable of protecting domestic pigs and wild suids against multiple ASFV genotypes [8,15].

Currently, live-attenuated vaccines (LAVs) represent the most promising approach for inducing protective immunity. Some LAV candidates, such as HLJ/18-7GD [157], appear to be safe for pregnant sows without noticeable effects on reproduction or offspring health [158]. Nevertheless, safety concerns remain with LAVs, including potential reversion to virulence, persistent infection, and recombination risk with field strains [159,160]. Regular monitoring of vaccine strains and the development of genetic safeguards are therefore important to reduce the risk of virulence reversion and to ensure the long-term safety of LAVs.

Alternative approaches, including viral-vectored, subunit, DNA, and mRNA vaccines, are also under investigation to improve safety and stability. For example, replication incompetent adenovirus-vectored vaccines expressing five immunogenic ASFV antigens have provided partial protection in pigs [161,162], and multi-antigen strategies using adenovirus and modified vaccinia Ankara vectors have shown protection against lethal challenge [162,163]. However, some studies using similar adenovirus vectors carrying ASFV genes failed to protect pigs when they were exposed to virulent genotype II strains, showing the difficulty to identify optimal antigen combinations [163]. More recent approaches using multicistronic cassettes spanning the entire ASFV Georgia 2007/1 proteome have shown promising protection, suggesting that multi-antigen formulations may enhance immune responses [164]. Advances in immuno-informatics and antigenic design multi-epitope vaccines and the use of purifying recombinant viruses such as CRISPR/Cas9 gene editing and efficient cloning systems are also accelerating ASF vaccine development [165,166,167]. These findings suggest that vectored vaccines are promising, but ASF immunization research is still developing. Despite these advances, most subunit vaccines still provide only partial or inconsistent protection, particularly against genotype II strains [168,169,170,171,172]. Integrating multi-antigen targets and improved delivery technologies such as nanoparticle-based platforms may enhance the effectiveness of subunit-based vaccines.

In wild suids, oral bait vaccine formulations containing gene-deleted ASFV strains have shown promising immune responses and acceptable environmental safety [173]. However, some approaches, such as the attenuated NH/p68 strain grown in MA104 cells, failed to provide sufficient protection, suggesting that vaccine formulation and delivery remain major challenges [174]. Therefore, future research should focus on optimizing bait formulations, improving vaccine stability in field conditions, and combining wildlife vaccination with effective surveillance and population management strategies.

Despite significant progress, a universally effective ASF vaccine is currently unavailable. Large-scale vaccination programs must consider cross-protection between virus genotypes, vaccine production capacity, and regulatory approval processes [175,176]. In this regard, the first ASF vaccine standards were recently adopted as part of the WOAH Terrestrial Manual in recognition of the development and commercialization of the first-generation ASF vaccine and for the purpose of providing minimum standards for safe and effective vaccines [177]. Importantly, vaccination alone cannot control ASF due to its complex transmission cycle; therefore, integrated strategies combining vaccination, strong biosecurity, surveillance, and wildlife management remain essential for effective disease control [3,68]. A detailed list of ASFV vaccines is given in Supplementary Table S2.

### 5.4. Biosecurity Measures

In the absence of a widely available and effective ASFV vaccine, control strategies heavily rely on the strict biosecurity implementation at farm, animal, and individual (personnel) levels.

At the farm level, biosecurity aims to prevent virus introduction through structural and management-based measures, including the separation of clean and dirty zones, controlled entry points, and perimeter fencing systems [178]. For instance, pig farms in Germany commonly implement designated hygiene barriers (anterooms) and farm zoning systems, although gaps in fencing and area separation still exists [178]. Double fencing systems are widely used in the European Union to minimize contact between domestic pigs and wild boar [81], whereas commercial farms in countries such as China [179] and the United States practice controlled feed supply chains, risk assessment scoring systems, and structured programs such as the Secure Pork Supply Plan to enhance biosecurity compliances [180]. Strong biosecurity and border controls underpin Australia’s prevention strategy for ASFV and other emerging animal diseases. Australian piggeries implement robust on-farm measures, including effective fencing, strict visitor and personnel protocols, surveillance and control of wild pig populations, and stringent feed regulations with a prohibition on swill feeding [181]. These are complemented by mandatory property registration and tight controls on animal movements to minimize the risk of disease introduction and spread.

At the animal level, biosecurity practices mainly focus on reducing disease transmission within and between pig groups through age or production stage segregation and batch management. Spot elimination approaches have also helped to reduce losses and protect farmer livelihoods [116]. In major pig-producing countries, the structured disposal of carcass and storage systems for manure management are commonly practiced [180].

At the individual level, human-mediated transmission could be mitigated through hygiene protocols such as dedicated farm-specific clothing and footwear, handwashing, the use of disinfectant footbaths, and controlled movement of staff between farm units [178,180,182,183]. High-biosecurity farms further implement strict entry protocols, including shower-in/shower-out systems, restricted access systems, visitor restriction, controlled access logs, and supervised entry procedures to reduce indirect transmission through visitors, veterinarians, and transport workers [178,184]. Despite these measures, ASFV continues to spread due to a lack of synchronization among farmers, government, public, and even nations to follow the precautionary measures systematically.

## 6. Major Challenges and Future Directions

### 6.1. Animal Movement

Animal movement is one of the major causes of ASF spread across regions and production systems. Long-distance dissemination of the virus can be facilitated by the movement of infected pigs and trade of live pigs, even before infected pigs begin showing clinical symptoms [185]. Furthermore, the virus may be introduced into previously disease-free areas through illegal trade and the uncontrolled movement of pigs, including movements associated with breeding or market supply chains, if proper biosecurity and quarantine are not maintained [186]. Higher risk of spread exists in the regions where pig production involves constant transport between farms, markets, and slaughterhouses [100]. In many developing countries, informal trade networks and live animal markets further increase the disease transmission chances as animals often move without proper health inspection [156]. Improving traceability systems, using digital identification for pigs, and applying movement permits can help authorities to better trace and detect outbreaks earlier. During an outbreak, combining real-time surveillance with movement control measures can also help to reduce the spread of disease.

### 6.2. Illegal Meat and Meat Products

The illegal trade and transportation of pork and pork products are major drivers of transboundary ASFV spread. Due to the environmental stability of ASFV, contaminated meat can remain infectious for prolonged periods [105]. The genetic material of ASFV is detected in illegally imported meat products, indicating the potential for virus introduction through travelers carrying contaminated pork and pork products, informal markets, and unregulated food trade [187,188]. Nevertheless, imported pork products may bypass veterinary inspection systems in several regions [189]. Domestic pigs and wild suids can become infected through the consumption of contaminated products that are improperly disposed or used as swill [190]. Therefore, improving border monitoring, food import regulations, public awareness campaigns, and rapid screening methods for imported animal products could reduce these risks since diagnostics are limited to risk-based inspections without ASFV specific testing. For example, in Australia, pork and pork products entering through airports or cargo channels undergo strict biosecurity controls administered by the Department of Agriculture, Fisheries and Forestry under the Imported Food Inspection Scheme. Meat products must be declared and screened using X-ray imaging, detector dogs, and manual baggage inspection [191]. Fresh pork is prohibited, whereas only certain processed products from approved countries are permitted with appropriate certification. In addition, commercial consignments undergo risk-based inspection and samples are tested for microbial contamination, chemical residues, and compliance with Australian food standards. On the other hand, non-compliant or undeclared items are seized and destroyed to prevent the introduction of exotic and economically important animal diseases such as ASF [192].

### 6.3. Human Movement

Farm workers, veterinarians, traders, and visitors can unintentionally spread ASFV on clothing, footwear, or farm equipment [78]. Additionally, international travel and labor migration, including backpackers or seasonal agriculture workers, may facilitate virus introduction through contaminated materials, food items or farm materials across borders [185]. A study in Asia demonstrated that the human-mediated movement of pigs, feed, and contaminated materials contributed to the rapid regional spread of the disease following virus introduction [115]. Significant reduction of such transmission requires robust training programs promoting hygiene protocols and on-farm biosecurity, strict visitor control policies, and raising awareness among travelers regarding contaminated animal products. In addition, farm-level biosecurity training programs for all personnel involved in pig production is also required. Effective control of ASFV depends on continuous and practical biosecurity training that improves farmers knowledge, attitudes, and compliance behaviors [178]. Similarly, community-based biosecurity programs in endemic regions such as Uganda demonstrated that awareness alone is insufficient unless training is adapted to socio-economic contexts and reinforces daily practices across the value chain [193]. Several countries and organizations have implemented structured awareness initiatives, including FAO-led training and e-learning programs for farmers, veterinarians, paraprofessionals, and hunters in Europe (2020–2025) that improved early detection, reporting, on-farm hygiene measures, and biosecurity in wild boar interfaces [194]. Likewise, Australia’s ASF prevention and preparedness project (initiated in 2020) reported that coordinated stakeholder engagement, training, and public awareness campaigns strengthen surveillance, early detection, and response capacity [195]. Therefore, integrated awareness strategies combining farm worker training, community engagement, and stakeholder education remain among the most effective non-pharmaceutical interventions for ASFV control.

### 6.4. Biosecurity Standards

Effective biosecurity implementation remains highly challenging due to structural, economic, behavioral, and epidemiological constraints. In farms, limitations in infrastructure, farm design, and financial capacity often hinder measures such as controlled entry points, sanitation barriers, and separation of clean and contaminated zones [105,196]. The complex epidemiology of ASFV further complicates control as the virus can persist in contaminated materials and the environment, whereas indirect transmission through fomites, feed, and human movement amplifies disease spread [8,11]. Control of wild and feral pig movement remain challenging due to their wide-ranging behavior and ability to cross diverse landscapes. Measures such as fencing and population control are difficult to implement at scale and are only partially effective. Therefore, coordinated surveillance and cross-border collaboration are essential. Emerging technologies, including AI-driven computer vision integrated with CCTV surveillance, may improve tracking of wild pig movement, whereas engagement of the hunting communities can support control efforts. Countries such as Australia have a dedicated feral pig control program [197]. Nevertheless, strategies should be tailored to regional geography and aligned with local environmental, cultural, and socio-economic conditions.

At the national level, biosecurity implementation is further challenged due to the lack of harmonized and enforceable standards. Although farms should adhere to standardized biosecurity protocols audited by national accreditation or veterinary authority, uniform compliance across diverse production systems may not be feasible, particularly among smallholder and backyard farms [198]. Economic barriers also discourage outbreak reporting and compliance with stamping-out policies when cost-sharing or compensation schemes are inadequate [199].

To overcome these challenges, strategies must be locally adapted, economically feasible, and socially inclusive. Biosecurity measures must be cost effective and context-specific, in combination with participatory approaches for smallholder farmers to adopt them with local practices and priorities [193,200]. Economic barriers can be addressed through fair compensation and cost sharing schemes for culled animals to encourage disease reporting and compliance. Future efforts should emphasize community-based biosecurity programs, interdisciplinary approaches integrating social and economic factors, and digital surveillance systems for sustainable ASF control.

### 6.5. Strict Border Security

The global spread of ASF over the past two decades highlights how international trade, weak border control, and inadequate monitoring systems have contributed to its rapid transboundary spread [201]. Strengthening border surveillance, the use of detector dogs for meat products, and increasing international collaboration on disease monitoring can enhance early detection and the prevention of ASF. All pork-producing countries should follow standardized biosecurity procedures formulated by international bodies such as the WOAH, with a strong focus on transboundary animal diseases. For instance, implementing strict biosecurity protocols at airports and seaports, supported by quarantine screening and robust enforcement measures are essential. These measures may include fines, visa cancellation, and deportation for failing to declare illegal meat and meat products. However, many countries still lack effective airport biosecurity programs, creating global gaps in ASFV prevention and control efforts.

### 6.6. Genetic Improvement of Pigs

Advanced technologies, including high-throughput genome sequencing and gene editing, are enabling the identification of candidate genes and the development of disease-resistant farm animals [202]. For instance, the porcine reproductive and respiratory syndrome virus (PRRSV) enters porcine cells via the CD163 receptor; accordingly, pigs genetically modified using CRISPR/Cas9 to delete CD163 exhibit resistance to PRRSV [203,204]. Although all wild-type pigs succumbed to infection, 75% of *CD163^Mut/Mut^* pigs survived and recovered, establishing a strong foundation for breeding PRRSV-resistant pigs through gene-editing technologies [205]. Furthermore, macrophages from genome-edited pigs lacking the CD163 SRCR5 domain are fully resistant to both American and European PRRSV genotypes while maintaining normal biological function [206]. Although CD163 plays a role in the permissiveness and infection process of porcine monocytes/macrophages by ASFV [207], pigs lacking CD163 did not exhibit resistance to the ASFV isolate Georgia 2007/1 [208]. Nevertheless, several studies have identified key host genes regulating ASFV permissiveness, including SLA-DM [209] and CRISPR/Cas9-edited germlines have been developed to evaluate the roles of these candidate genes [210]. A CRISPR/Cas9 knockout study also identified TMEM239 as an important host factor facilitating ASFV entry into early endosomes [211]. Encouragingly, multiple research groups are working to identify genes associated with ASFV infection and host tolerance [212]. Comparative genomic studies further indicate that genetic variation among pig breeds influences ASFV infection rates [213,214]. Understanding the function of host genes is crucial, and advanced biotechnology such as CRISPR/Cas9 may help unravel their roles. For instance, CRISPR/Cas9-mediated editing has identified that the T-cell receptor beta (TRBV27) CDR1 sequence is associated with ASFV infection in pigs [215]. The application of advanced breeding technologies based on gene editing holds strong promise to transform the pig industry by improving animal health and productivity [216].

### 6.7. Policy Development and Implementation

Effective policy frameworks are essential for ASF control and preparedness. However, many countries face difficulties implementing coordinated control strategies due to limited resources, inconsistent regulations, and weak enforcement mechanisms [217]. Furthermore, differences in national disease control policies, reporting systems, and compensation schemes may hinder rapid outbreak responses and cooperation between stakeholders [218]. In some regions, insufficient policy guidance for smallholder pig systems further complicates disease control efforts [156]. Therefore, policy makers should focus on developing coordinated international policies, better compensation schemes, and stronger One Health-based surveillance systems, integrated risk assessment frameworks, and emergency preparedness plans to enhance ASF prevention and outbreak responses.

### 6.8. Global Coordination

**Figure 6:**
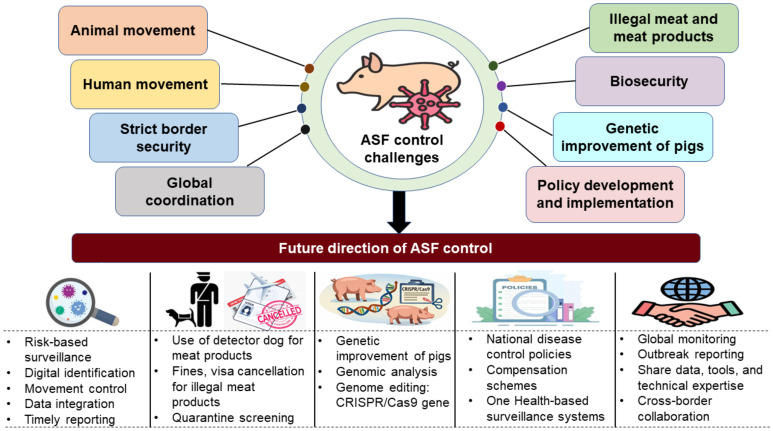
ASFV prevention and control challenges.

Global coordination is critical for effective ASF control as the disease spreads across national borders and requires harmonized international action. Existing global bodies such as the World Organization for Animal Health (WOAH), Food and Agriculture Organization (FAO), and Global Framework for the Progressive Control of Transboundary Animal Diseases (GF-TADs) provide a foundation for standards setting, coordinated global monitoring, outbreak reporting, and response systems, enabling countries to share surveillance data and align control strategies. The Global African Swine Fever Research Alliance (GARA) is an international network established in 2013 which brings researchers together to generate knowledge and practical solutions to monitor and control ASF worldwide [219]. Ongoing support for research networks such as GARA will be critical for information sharing, fostering collaboration and the identification of knowledge and technological gaps for supporting the prevention, control, and eradication of ASF. Since ASF transmission patterns vary across regions due to differences in socio-economic conditions, husbandry practices, trade activities, border control, and biosecurity practices, strong, multifaceted collaborations between government and private sectors are needed to enforce biosecurity across the value chain. The major challenges for prevention and control of ASF globally are summarized in Figure 6.

## 7. Conclusions

The evolving challenges of ASFV are reflected in its expanding global epidemiology, persistent transboundary spread, recurrent outbreaks, and a complex multi-host transmission cycle involving domestic pigs, wild boar, and human-mediated pathways. Epidemiological evidence suggests that indirect transmission through contaminated fomites, pork products, and the movement of infected animals plays a dominant role in long-distance spread, whereas wild boar populations serve as long-term wildlife reservoirs that sustain endemicity in several regions. A deeper understanding of ASFV biology, particularly host–virus interaction and immune evasion mechanisms, is essential to support rational vaccine design. However, the development and deployment of vaccines requires robust post-vaccination safety and efficacy monitoring, including surveillance for adverse effects, field effectiveness, and potential virus evolution under immune pressure. Until safe and widely effective vaccines are achieved, control strategies must prioritize the strengthening of disease management and risk-based surveillance systems, early detection, and strict on-farm biosecurity. Critical measures include minimizing contact between wild and domestic pigs, banning swill feeding, vector control in specific ecological settings where soft ticks contribute to transmission cycles, regulating animal movement, carcass removal, and reinforcing border biosecurity. Importantly, these interventions must be economically feasible and practically applicable, requiring strong coordination among governments, private sectors, farmers, and veterinarians. Sustained progress depends on effective policy implementation and international coordination and data sharing among veterinary authorities, WOAH, FAO, researchers, and the livestock industry to ensure harmonized outbreak reporting, and cross-border control. Overall, a coordinated approach integrating enhanced surveillance, strict farm biosecurity and border security, structured wild boar management, cautious vaccine development and monitoring, and strong international collaboration is required to mitigate the global impact of ASFV on pig health and the livestock industry.

## Figures and Tables

**Table 1:** Different layers of the ASF virion with functions of constituent proteins.

Viral Components	Genomic Coding Region	Proteins Involved in Assembly	Functions and Features of the Proteins	References
Outer membrane/envelope	061R	p12	Aids in viral adsorption by facilitating the recognition of host cellular receptors through specific polypeptide binding and ensures virus entry into the host cell	[22]
	EP402R	CD2v	Interacts with sialic acid receptors on RBC surfaces, enabling infected cells to bind to them and accelerates hemadsorption	[23]
	KP177R	p22	Unknown	[24]
Capsid	B646L	p72 (major capsid protein)	Key antigen for serological diagnosis due to its antigenic stability and strong immunogenicity. It also plays role in morphogenesis, replication, and cellular transport	[25,26]
	B602L	pB602L (chaperone of p72)	This molecular chaperone assists correct folding and trimerization of p72 during capsid assembly	[27]
	H240R	pH240R	Essential for virulence and pathogenicity, triggering infection-induced inflammatory responses	[28,29]
	M1249L	pM1249L	Functions in immunomodulation	[30]
	D117L	p17	Binds strongly to the p72 base domain, surrounding each p72 capsomer within the inner capsid shell. This strong binding anchors the virus to the inner membrane, which is crucial for its stability and viability. p17 is vital in capsid assembly and maturation	[31,32]
	E120R	p14.5	Interacts with p72 and plays a crucial role in transporting mature virus virions from the viral factory to the host cell membrane, helping in virion release and subsequent host infection	[33]
	B438L	p49	Plays an important role in morphogenesis, particularly in the formation of the icosahedral capsid vertices	[34]
Inner membrane/ envelope	E183L	p54	Enables viral entry into the perinuclear region by interacting and forming microtubule-based motor complexes. It also acts as a diagnostic antigen to detect antibodies of ASFV	[35,36]
	E248R	pE248R	Required for virus–cell fusion, early infectivity, and viral core transfer to the cytoplasm	[37]
	H108R	pH108R	Interacts with capsid proteins p49 and p72 and helps in capsid assembly. It also has a regulatory role in ASFV morphogenesis. Deletion of pH108R reduces virus replication and attenuates ASFV virulence	[38]
	E199L	pE199L	Necessary for membrane fusion, viral uncoating, and viral core entry	[39]
Core shell	S273R	pS273R	Essential for virion maturation and infectivity of the ASFV particle	[40]
	CP2475L (pp220 polyprotein)	p150	Highly immunogenic	[22]
		p37	Aids viral entry and accumulation in the cytoplasm by facilitating transport between the nucleus and cytoplasm	[6]
		p34	Highly conserved and immunogenic. It contains a T-cell epitope, indicating its potential to trigger a cell-mediated immune response. Mutations in the p34 are linked to replication and assembly of the ASFV virion	[41]
		p14	Promotes nuclear transport activity by aiding virus replication	[6]
		p5	Encodes a unique tryptic peptide covering 43% of its amino acid sequence	[42]
	CP530R (pp62 polyprotein)	p15	Interacts with other components during viral assembly and aids in the stabilization of the mature virus particles. It may also be involved in viral transcription and genome packaging by binding with dsDNA	[43]
		p35	Serves as a docking scaffold, helping to recruit the host membrane and other components to the core shell, thus stabilizing the mature virion during assembly	[4]
		p8	Low immunogenicity and rapid degradation	[42]
Inner core/Nucleoid	A104R	pA104R (histone-like protein)	Induces DNA supercoiling and plays a role in genome packaging during virus assembly and virus evasion of host immunity	[15,44,45]
	K78R	p10	It has a similar affinity for both dsDNA and ssDNA and supports viral replication, entry, and DNA packing	[25,46,47]

## Data Availability

No new data were created or analyzed in this study.

## Supplementary Materials

The following supporting information can be downloaded at: https://www.mdpi.com/article/10.3390/pathogens15060586/s1, Table S1. Chronological summary of ASFV outbreaks worldwide [4,8,10,13,17,68,94,95,97,98,100,101,103,105,109,110,111,113,116,117,122,125,133,220,221,222,223,224,225,226,227,228,229,230,231,232,233,234,235,236,237]; Table S2. List of ASFV vaccines with their types, strain used, developers and manufacturers (where relevant), efficacy, side effects and limitations [158,161,165,168,170,172,238,239,240,241,242,243,244,245,246,247,248,249,250,251,252,253,254,255,256,257].
