# Supplementary material for: Current Insights into the Epidemiology and Transmission Dynamics of African Swine Fever Virus and Future Control Perspectives
Source: Pathogens. 2026 May 29;15(6):586. doi: 10.3390/pathogens15060586 (PMC13306203; doi:10.3390/pathogens15060586)
Supplement: Supplementary file 1 [file pathogens-15-00586-s001.zip › pathogens-4323966-supplementary.pdf]

Supplementary Table S1. Chronological summary of ASFV outbreaks worldwide

Year	Country/Region	Host	Disease spread/ Re-emergence	References
1909	Kenya	Domestic pigs (Spillover from wildlife)	First description of disease	[94]
1921	East Africa (Kenya)	Wild suids and soft ticks (*Ornithodoros moubata*)	Endemic	[68,94,95]
Late 1920s-early 1930s	North-Eastern regions of South Africa, Angola, and Malawi	Domestic pigs, warthogs, and soft ticks (*Ornithodoros* spp.)	Southward spread from East Africa (endemic in southern Africa) and regional spread in sub-Saharan Africa	[8,97,98,105]
1930-ongoing	Sub-Saharan Africa	Domestic pigs and wild reservoirs	Endemic persistence	[98,100]
1957	Portugal (Lisbon)	Domestic pigs	First outbreak outside Africa	[10,101]
1960	Spain and Portugal	Domestic pigs and tick cycle (*O. erraticus*)	Emergence/re-emergence	[10]
1964-1986	France, Italy, Malta, Belgium, the Netherlands	Domestic pigs	Spread across Europe	[10,17,101,103]
1967-1995	Italy (Sardinia)	Domestic pigs, wild boar, and ticks	Persistent (long-term endemic)	[107]
1971	Cuba	Domestic pigs	First introduction to Americas	[10,17,101,103]
1978-1980	Brazil, Dominican Republic, Haiti	Domestic pigs	Regional spread, eradicated	[10,17,101,103]
1996	Côte d'Ivoire	Domestic pigs	Eradicated by early 1997	[220,221]
1997	Benin, Togo, Nigeria	Domestic pigs	Transboundary regional epidemic	[221]
1998	Madagascar	Domestic pigs	Endemic	[222-224]
1999	Ghana	Domestic pigs	Eradicated in 2000; re-introduced in 2002	[221,225,226]
1999	Portugal	Soft tick (*O.erraticus*)	Endemic	[227]
2003	Burkina Faso	Domestic pigs	Persisted after re-introduction in 2002	[221]
2007	Georgia (Caucasus)	Domestic pigs, wild boar	Emergence; beginning of Eurasian panzootic	[4,13,111]
2007-ongoing	Russia, Armenia, Belarus	Domestic pigs, wild boar	Rapid regional spread	[8,105,228]

2014	Baltic States (Lithuania, Latvia, and Estonia) and Poland	Domestic pigs, wild boar (major reservoir)	First EU wild boar driven epidemic	[109,110,229,230]
2018	China	Domestic pigs	Emergence (First detection in Asia)	[113,231,232]
2019-2024	Mongolia, Democratic People's Republic of Korea, Hong-Kong, Bhutan, Republic of Korea, the Philippines, Singapore, Malaysia, Indonesia, Timor-Leste, Papua New Guinea, Vietnam, Lao People's Democratic Republic, Cambodia, Thailand, Myanmar, Bangladesh, Bhutan, Nepal, India, Sri-Lanka	Domestic pigs	Regional epidemic across Asia and expansion in the Indian ocean region	[70,116,117,120,125,233,234]
2020	Germany	Wild boar	Re-emergence (Western Europe); wildlife-driven persistence	[109,110]
2021	Dominican Republic, Haiti	Domestic pigs	Re-emergence, later eradicated	[122]
2025-2026	Bosnia and Herzegovina, Croatia, Latvia, Moldova, Romania, Serbia, Republic of Korea, India, the Philippines	Domestic pigs	Ongoing	[133,235,236]
2025-2026 (Up to March)	Hong-Kong, Bulgaria, Estonia, Poland, Germany, Greece, Latvia, Hungary, Italy, Lithuania, Moldova, Romania, Poland, Spain, Ukraine, Croatia, Serbia, Slovakia	Wild boar	Ongoing	[133,235-237]

Table S2. List of ASFV vaccines with their types, strain used, developers and manufacturers (where relevant), efficacy, side effects and limitations.

Category	Vaccine/ Candidate	Vaccine Type	Strain/ Genetic Basis	Developer/ Manufacturer	Genotype Coverage (I-XXIV)	Reported efficacy	Side effects/ Limitations	References
Licensed, emergency/field use vaccine	ASFV-G-ΔI177L	Live attenuated	Georgia 2007/1 lineage (with I177L deletion)	Developer: U.S. Department of Agriculture-Agriculture Research Service (USDA-ARS). Manufacturer: NAVETCO (Vietnam)	Genotype II (limited evidence on genotype I)	Strong protection: homologous strains; partial cross protection	Residual virulence, heterogenous protection: limited	[238,239]
	ASFV-G-ΔMGF	Live attenuated	Georgia strain with	Developer: USDA-ARS.	Genotype II	Strong protection: homologous strains	Chronic infection, fever, poor cross	[172,238]

			MGF360/505 deletions	Manufacturer: AVAC Vietnam Joint Stock Company			protection beyond closely related strains	
Experimental	BA71ΔCD2	Recombinant live attenuated prototype	BA71 strain with CD2v deletion	IRTA-CReSA (Spain)	Genotype I	Cross protection: some genotype II strains	Mild, transient viremia, vaccine virus in blood and nasal secretion (limited shedding)	[240]
	HLJ/18-7GD	Live attenuated	HLJ/18 strain (7-gene deletion)	Chinese Academy of Agricultural Sciences (CAAS) (China)	Genotype II	Homologous protection; safe in pregnant sows	Mild fever, joint swelling, risk of reversion, heterogenous genotypes: no solid protection	[158,241]
	SY18 mutants	Live attenuated	China SY18 strain mutants (ΔI226R/ ΔCD2v)	Harbin Veterinary Research Institute (China)	Genotype II	Strong protection: homologous strains	Safety concern: incomplete attenuation, Cross-protection: limited	[242-244]
	JX23-02D250R (HYBRID I/II)	Live attenuated	Virulent genotype I/II hybrid (gene deleted)	CAAS with Jilin University and other institutions (China)	Genotype I/II recombinant	Infection with both the homologous parenteral I/II recombinant strain and heterogenous: 100% survival	Cross protection: limited against multiple genotypes	[245]
	Chemically inactivated ASFV (CT-ASFV)	Inactivated whole-virus vaccine	Bali isolate B1	Faculty of Veterinary Medicine, Udayana University; Disease Investigation Center, Indonesia	Genotype II	67% survival/protection after testing	Only homologous virulent challenge, partial protection	[246,247]
	Binary ethyleneimine (BEI) chemically inactivated vaccine	Inactivated whole virus (β propiolactone/BEI-type inactivated approach)	Homologous virulent challenge strain	VISAVET Centre at the Complutense University of Madrid	Genotype II	Induced ASFV-specific antibodies, safe at high doses,	No major adverse reactions, no cross protection	[248]

Gamma irradiation inactivated ASFV	Physically inactivated whole virus (gamma irradiation)	Vaccine strain: ASFV Estonia 2014 Challenge strain: ASFV Armenia 2008	Friedrich-Loeffler-Institu (FLI), Germany and Animal Production and Health Laboratory of the International Atomic Energy Agency (IAEA) in Austria	Genotype II	Induce p72-specific IgG antibodies	All animals developed acute lethal ASF within 6 days, post-challenge fever, no protection after virulent challenge	[249]
Subunit vaccines (p30, p54, p72)	Subunit; Clinical challenge trials	Antigens from Genotype I/II	JinYu Bio-Technology Co., Ltd. (China) and other multiple institutes	No reliable protection across genotypes	Immunogenic but not fully protective	Lack in cellular immunity	[170,250]
DNA/ Viral vector vaccines	DNA/vectored	Cocktail of eight viral vectors (Adenovirus and MVA)	Shandong University, China; Agricultural Research Service (ARS), Pirbright Institute (UK) etc.	Genotype coverage: Inconsistence	100% protection against lethal challenge in experimental studies	Heterogenous strains: limited protection	[161,168,251]
p54 LNP-DNA vaccine	Plasmid DNA vaccine (lipid nanoparticle-encapsulated)	ASFV p54 gene (E183L antigen)	University of Nebraska-Lincoln (NU)	Likely genotype II	Induced strong antibody and T-cell responses	No protection efficacy confirmed (no challenge study)	[252]
Multi-epitope ASFV DNA vaccine constructs	Plasmid DNA vaccine (in silico designed, cloned in pVAX1)	p72 (B646L), p30 (CP204L), p54 (E183L), CD2v (EP402R)	Chulalongkorn University in Thailand	Broad (I-XXIV, predicted)	In silico strong immunity	No in vivo validation, real-world immunogenicity and protection unknown	[165]
Multi-antigen DNA constructs	Plasmid DNA vaccine	CD2v-like proteins, p30, p54, p72	American Chemical Society (ACS) Infectious Disease study	Cross-genotype potential	Immunogenic	No consistent protection across ASF challenge models	[253]
Multi-antigen mRNA vaccine	multi-antigen cocktail	CD2v (EP402R), EP153R, p30 (CP204L), p54 (E183L), p72 (B646L), B602L	University of Science and Technology School of Medicine in Shenzhen, China	Potential cross-genotype (especially genotype II)	Strong immunity	Protection efficacy still limited	[254]

	Subunit mRNA cocktail	mRNA vaccine	Rationally selected ASFV antigens	Koch Institute for Integrative Cancer Research at the Massachusetts Institute of Technology (MIT) with other collaboration	Broad (potential cross-genotype)	Immunogenic	Protection not demonstrated, requires further validation	[253]
Vaccine-like or poorly attenuated strains	Naturally attenuated isolates (e.g: OURT88/3)	Not applicable (N/A)	OURT88/3	Pirbright Institute (UK), CISA-INIA (Spain), University of Lisbon (Portugal)	Genotype I	Homologous virulent challenge: High protection	Residual virulence, transient fever, arthritis, skin necrosis, retarded growth, pneumonia	[255-257]
